# Stochastic simulations of minimal cells: the Ribocell model

**DOI:** 10.1186/1471-2105-13-S4-S10

**Published:** 2012-03-28

**Authors:** Fabio Mavelli

**Affiliations:** 1 Chemistry Department, University "Aldo Moro", Bari, 70125, Italy

## Abstract

**Background:**

Over the last two decades, lipid compartments (liposomes, lipid-coated droplets) have been extensively used as in vitro "minimal" cell models. In particular, simple and complex biomolecular reactions have been carried out inside these self-assembled micro- and nano-sized compartments, leading to the synthesis of RNA and functional proteins inside liposomes. Despite this experimental progress, a detailed physical understanding of the underlying dynamics is missing. In particular, the combination of solute compartmentalization, reactivity and stochastic effects has not yet been clarified. A combination of experimental and computational approaches can reveal interesting mechanisms governing the behavior of micro compartmentalized systems, in particular by highlighting the intrinsic stochastic diversity within a population of "synthetic cells".

**Methods:**

In this context, we have developed a computational platform called ENVIRONMENT suitable for studying the stochastic time evolution of reacting lipid compartments. This software - which implements a Gillespie Algorithm - is an improvement over a previous program that simulated the stochastic time evolution of homogeneous, fixed-volume, chemically reacting systems, extending it to more general conditions in which a collection of similar such systems interact and change over the course of time. In particular, our approach is focused on elucidating the role of randomness in the time behavior of chemically reacting lipid compartments, such as micelles, vesicles or micro emulsions, in regimes where random fluctuations due to the stochastic nature of reacting events can lead an open system towards unexpected time evolutions.

**Results:**

This paper analyses the so-called Ribocell (RNA-based cell) model. It consists in a hypothetical minimal cell based on a self-replicating minimum RNA genome coupled with a self-reproducing lipid vesicle compartment. This model assumes the existence of two ribozymes, one able to catalyze the conversion of molecular precursors into lipids and the second able to replicate RNA strands. The aim of this contribution is to explore the feasibility of this hypothetical minimal cell. By deterministic kinetic analysis, the best external conditions to observe synchronization between genome self-replication and vesicle membrane reproduction are determined, while its robustness to random fluctuations is investigated using stochastic simulations, and then discussed.

## Background

In recent years, many researchers have been actively working in the field of the *de novo *synthesis of the artificial cell [1-5], i.e. a cell made from scratch using both synthetic and natural compounds. This scientific challenge has many relevant aspects: first of all, it can reinforce the theory of abiogenesis in the origins of life debate [6], proving that life can emerge spontaneously in a test tube, at least in suitable experimental conditions. Furthermore, the possibility to create a population of artificial cells programmed for the synthesis of chemical compounds of pharmacological and industrial interest is a significant biotechnological goal [7,8]. Artificial cells can also be envisaged, in the maybe not too distant future, as microscopic diagnostic and pharmacological labs to be delivered into the human body in order to synthetize and release drugs as a response to an external stimulus in presence of a disease [9].

The topic of artificial cells is strongly related to the minimal cell notion that defines the simplest cell to be considered alive and then to be experimentally implemented. A minimal living cell, or protocell, can be defined as the minimum supramolecular bounded structure based on the lowest number of molecular species and metabolic processes that is capable of self-maintaining, self-reproducing and evolving [10]. *Self-Maintenance *is the necessary condition that must be fulfilled by the protocell to be considered alive, i.e. it must stay in a steady state where all its constituents are continuously synthesized and refurbished [11]. Nevertheless, this is not sufficient to implement cellular life as we actually know it; in fact, real cells are also able to grow and to self-reproduce. On the other hand, *Self-Reproduction *can be seen as a consequence of the cellular metabolism that can keep the minimal cell in a stationary state or bring it to a continuous growing regime that forces the organism to divide in order to maintain its internal coherence, i.e. its stability. Both these features are individual properties of a single cell that can be observed during its life time. In contrast, *Evolvability *is a collective property that can be exhibited only by a population of cells and on a time scale of several generations, according to a Darwinian selection mechanism [6,12].

Some years ago, Szostak and colleagues proposed a minimal cell prototype called the *Ribocell*: the RNA-based cell, that in principle can exhibit all the three properties to be considered a living protocell [13]. This theoretical cellular model consists in a self-replicating minimum genome coupled with the self-reproduction of the lipid vesicular container [14]. These authors envisaged the existence of two hypothetical ribozymes [13], one (R_L_) able to catalyze the conversion of molecular precursors (P) into membrane lipids (L) and the other one (R_P_) able to duplicate RNA strands. Therefore, in an environment rich in both lipid precursors (P) and activated nucleotides (NTPs), the Ribocell can self-reproduce if both processes, i.e. genome self-replication and membrane reproduction (growth and division), are somehow synchronized.

**Figure 1:**
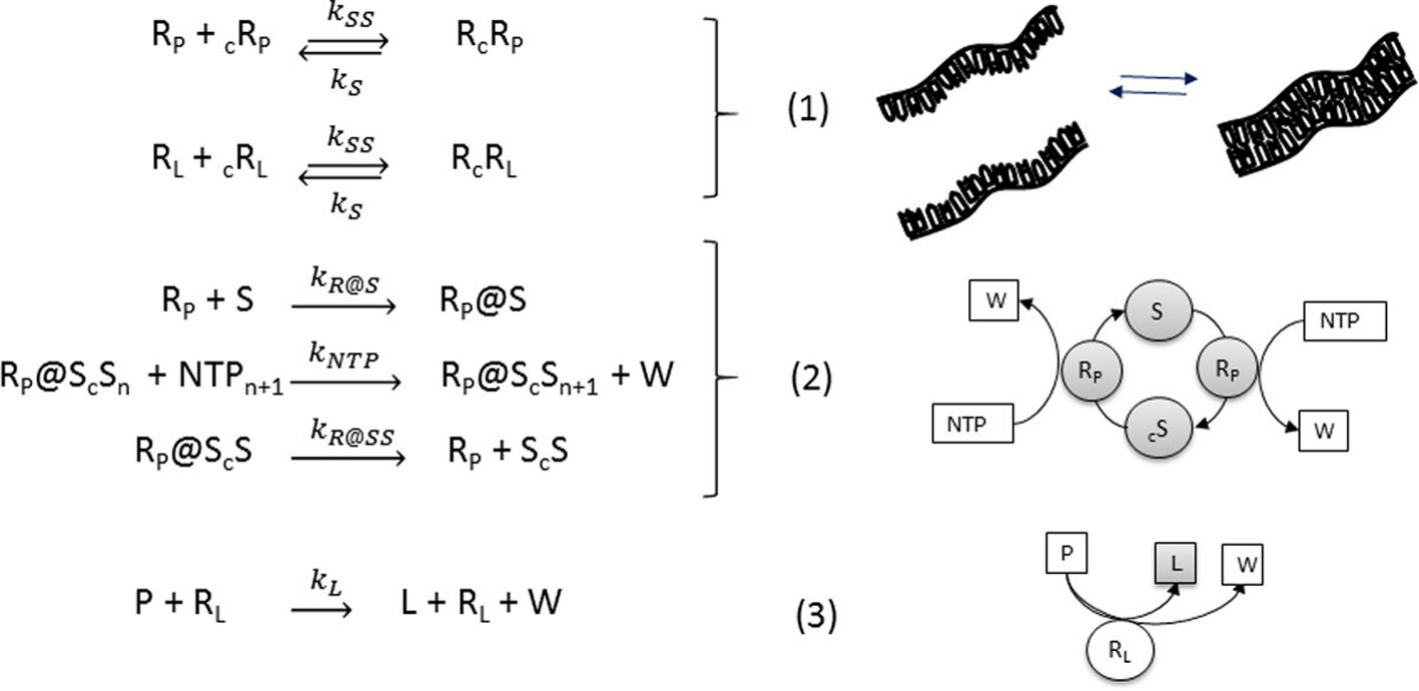
**The Ribocell internal metabolism**. (1) Reversible RNA strand association, (2) catalyzed template transcription (S = R_P_, _c_R_P_, R_L_, and _c_R_L_,) (3) lipid synthesis.

In previous papers [15,16], we have presented an *in silico *implementation of the Ribocell based on the internal metabolism reported in Figure 1 and on the recently introduced self-replicating lipid vesicle model [17]. By means of a deterministic analysis, we showed that if the kinetic constant for lipid formation *k_L _*is in the range: 1.7·10^3^s^-1^M^-1^≤*k*_L_≤1.7·10^5^s^-1^M^-1 ^then synchronization between vesicle reproduction and genome replication can spontaneously emerge under the model assumptions and kinetic parameters reported in Table 1. Deterministic calculations were performed for two ribozymes 20 bases long and showed that the Ribocell reaches a stationary growth and division regime, where the cell size remains constant after each division along with the amount of genetic materials. Although the observed cell life time stabilizes after the first 10 generations, it remains very high, at over 80 days for all the *k_L_*values in the synchronization range, making the Ribocell very hard to implement and study experimentally.

**Table 1:** Kinetic Parameters for the *in silico *Ribocell model at room temperature.

Kinetic Parameters	Values	Process Description	**Ref**.
*k*_SS_[s^-1^M^-1^]	8.8·10^6^	Formation of dimers R_c_R_P _and R_c_R_L_	[28]
*k*_S_[s^-1^]	2.2·10^-6^	Dissociation of dimers R_c_R_P _and R_c_R_L_	[28]
*k*_R@S_[s^-1^M^-1^]	5.32·10^5^	Formation of R@S	[29]
*k*_R@SS_[s^-1^]	9.9·10^-3^	Dissociation of Complexes R@S_c_S	[29]
*k*_NTP_[s^-1^M^-1^]	0.113	Nucleotide Polymerization in Oleic Vesicle	[31]
*k*_L _[s^-1^M^-1^]	1.7·10^3^	Lipid Precursor Conversion*	[30]
*k*_in _[dm^2^s^-1^]	7.6·10^19^	Oleic acid association to the membrane	[26]
*k*_out _[dm^2^s^-1^]	7.6·10^-2^	Oleic acid release from the membrane	[26]
*P*_P _[cm·s^-1^]	4.2 10^-9^	Membrane Permeability to Lipid Precursor	
*P*_NTP _[cm·s^-1^]	1.9 10^-11^	Membrane Permeability to Nucleotides	[31]
*P*_W _= *P*_S_	0.0	Membrane Permeability to W and genetic staff	
*P*_aq_[cm·s^-1^]	1.0·10^-3^	Oleic Acid Membrane Permeability to Water	[32]

*k_L _is 10^5 ^times larger than the value of the splicing reaction, catalyzed by the hammerhead ribozyme.

In this paper, we first apply this model to 100-base-long ribozymes, in an attempt to find the best experimental conditions to reduce so as Ribocell life time. By using the deterministic approach, the robustness of the stationary growth and division regime will be investigated in terms of external substrate concentrations, vesicle size and initial ribozyme amount in order to define optimal external conditions for Ribocell self-reproduction.

Therefore, the influence of ribozyme length will also be explored in the optimal external conditions by ranging strand size from 20 to 200 bases in length and keeping all the other kinetic parameters constant. 20 bases is in fact the minimum length required to observe a folded RNA structures, i.e. a structure that can reasonably exhibit catalytic action. On the other hand, entities of about 200 nucleotides have been suggested as plausible ancient proto-ribosomes [18] even though, more recently, smaller subunits of 60 nucleotides have also been considered as plausible candidates [19]. Moreover, the dependence of Ribocell time behavior on the kinetic constants of RNA dimer formation and dissociation will also be studied.

Finally, stochastic simulations will be performed in order to test the robustness of the ribocell base on 100-base length ribozymes in optimal external conditions, with the aim of elucidating the role of intrinsic and extrinsic stochasticity on the time behavior of a protocell population.

## Methods

Self-reproducing vesicles are compartmentalized chemically-reacting systems where self-assembly processes are coupled with chemical reactions that produce amphiphilic molecules. To study the time behavior of these systems, we use both a deterministic and a stochastic approach in order to get insights into the average behavior of the protocell population and, at the same time, to elucidate the role of random fluctuations. Given a certain minimal cell model, i.e. a reaction mechanism with all the required parameters (kinetic constants, permeability coefficients, initial concentrations), the deterministic analysis can be done by numerically solving the ordinary differential equation set (ODES) or by analytically integrating an approximated reduced set of differential equations. Examples of the latter approach can be found in our previous works where self-reproducing micelles [20] and vesicles [21] were studied. In order to take into account the stochastic effects, some years ago we developed a Monte Carlo program [22] based on the Gillespie's direct method [23] designed for coupling amphiphile self-assembly [24] with chemical reactions and hydrophobic solute absorption [25] in a homogeneous well stirred macroscopic reactor. More recently, this program has been improved and a computational platform called ENVIRONMENT [26] was later developed to cope with the more general case of a collection of reacting lipid compartments that interact and change over time. In the next sub-sections, the general model for a self-reproducing vesicle will be recalled and discussed [17].

### *In silico *chemically reacting vesicles

A chemically reacting vesicle can be described as a homogeneous reacting aqueous domain enclosed by a lipid bilayer. Molecules can be exchanged with the external environment thanks to transport processes through the lipid membrane. A flux of water can also take place through the membrane in order to balance the osmotic pressure, i.e. the difference between the internal and the external overall concentrations. Chemical reactions can occur in the vesicle water core, according to the assumed internal metabolism, and amphiphilic molecules can be absorbed from and released towards both the external and internal aqueous solutions. Hence, the vesicle time state is defined by the following array:

(1) $$x^{\text{T}}=(n_{1}{}^{\text{C}},...,n_{N}{}^{\text{C}},n_{\text{L}}{}^{\mu },V_{\text{C}})$$

where *n*_i_^C ^are the molecular numbers of species X_i _(*i *= 1,2 ... *N*) present in the vesicle aqueous core and *n*_L_^μ ^is the number of amphiphiles X*_L _*(1≤ *L *≤*N*) in the membrane. *V*_C _is the water internal volume. In the stochastic approach, all *n*_i_^C ^and *n*_L_^μ ^are discrete integer numbers and there exist as many arrays as vesicles in the systems, while in the deterministic analysis there is a single array with real values that represents the average time state of the entire reacting vesicle population.

Table 2 compares the concentration change rates with the event propensity probability density functions for all the mentioned processes. The deterministic reaction rates are given according to the mass action law and they make it possible to write down the deterministic set of ordinary differential kinetic equations. On the other hand, the propensity probability density functions are used to carry out Monte Carlo simulations according to the Gillespie's direct method by means of the ENVIRONMENT software [26]. Moreover, in the stochastic simulations, the water flux will be considered instantaneous due to the high permeability of lipid membranes to water. At each step throughout the MC run, the aqueous vesicle core volume is rescaled in order to keep the vesicle in an osmotic balanced state by using the formula reported in Table 2. Conversely, deterministic calculation will explicitly take into account the water flux dynamics through the lipid membrane. However, a discrepancy between the two approaches can emerge only in presence of an abrupt change of the osmolite external concentration, due to fast dilution or solute addition of the external solution [26]. Another main difference between the two approaches is how the encapsulated molecules are distributed between the daughters after protocell division. In the deterministic approach, all the molecular content of the mother vesicle is halved while it is randomly distributed between the twin daughters in the stochastic simulations.

**Table 2:** Deterministic rates against propensity density probability for reacting and transport events.

Event	Deterministic Rate (*Ms*)^-1^	Propensity Density Probability *s*^-1^
Internal Chemical reactions ^(a)^ $a_{1,\rho }X_{1}+\ldots a_{N,\rho }X_{N}\overset{r_{\rho }}{\underset{}{\to }}b_{1,\rho }X_{1}+\ldots b_{N,\rho }X_{N}$	$k_{\rho }\overset{N}{\underset{j}{\prod }}{\left(\frac{n_{j}^{C}}{V_{C}N_{A}}\right)}^{a_{j},\rho }$	$\frac{k_{\rho }}{{\left(V_{C}N_{A}\right)}^{M_{\rho }-1}}\overset{N}{\underset{j}{\prod }}\left(\begin{array}{c} n_{j}^{C}\\ a_{j,p} \end{array}\right)$
Solute X_n _membrane transport ^(b)^	$P_{n}\frac{S_{\mu }}{V_{C}}\left(C_{n}^{E}-C_{n}^{C}\right)$	$D_{n}S_{\mu }{\frac{\left|\left(C_{n}^{E}-C_{n}^{C}\right)\right|}{\lambda_{\mu }}}^{\left(c\right)}$
Membrane Lipid Release	$\frac{k_{out}n_{L}^{\mu }}{N_{A}V_{C}}$	$k_{out}n_{L}^{\mu }$
Membrane Lipid Uptake	$\left(\frac{k_{in}S_{\mu }\left[X_{L}^{C}\right]}{N_{A}V_{C}}\right)$	$k_{in}S_{\mu }\left[X_{L}^{C}\right]$
Water Flux ^(d)^	$v_{aq}P_{aq}S_{\mu }\left(C_{T}^{E}-C_{T}^{C}\right)$	$V_{C}=\overset{N}{\underset{i=1}{\sum }}n_{i}^{C}/\left(N_{A}C_{T}^{E}\right)$

^a)^**a **and **b **stoichiometric matrixes, *N*_A _Avogadro's number, *k*_ρ _kinetic constant, *M*_ρ_molecularity [22]

^(b) ^The relationship between the macroscopic permeability *P*_n _and the molecular diffusion coefficient *D*_n _is: *D*_n _= *P*_n_λ_μ_*N*_A_, λ_μ _being the membrane thickness.

^(c) ^The absolute value guarantees that the propensity density probability is positive and the molecules move in the opposite direction from the concentration gradient.

^(d)^*v*_aq _is the water molar volume, while *C*_T_^E ^and *C*_T_^C ^are the total osmotic concentration in the external and internal aqueous solutions, respectively.

### Self-reproducing vesicles

The vesicle surface is estimated by the formula: S*_μ _*= (*α_L_*n*_L_^μ^*)/2 where *α_L_*is the amphiphile X*_L _*head area and 1/2 takes into account the double layered structure of the membrane. In presence of the synthesis of fresh amphiphiles, the membrane surface and the aqueous core volume can follow two different dynamics and this may bring the vesicles towards unstable conditions. The stability of the vesicle membrane can then be monitored by means of the reduced surface *ϕ*:

(2) $$\phi =\frac{S}{S_{V_{C}}^{\emptyset }}=\frac{S_{\mu }}{\sqrt[{3}]{36\pi V_{C}^{2}}}$$

that is, the ratio between the actual membrane surface *S*_μ _and the spherical area that would perfectly wrap the actual core volume V*_C_*. Assuming that, for a given size, the spherical shape (*ϕ *= 1) represents the minimum energy state, swollen (*ϕ *< 1) and deflated (*ϕ *> 1) vesicles are in high energetic conditions due to the elastic and the bending tension, respectively. Therefore, vesicles are assumed to be stable only in a small range of *ϕ *values around 1:

(3) $$\left(1-\varepsilon \right)\leq \phi \leq 2^{1/3}\;\left(1+\eta \right)$$

*ε *and *η *being the osmotic and dividing tolerance coefficients, respectively. In fact, vesicles in hypotonic solutions can swell, stretching the membrane until they reach a critical state: *ϕ *= 1-*ε*. The osmotic tolerance *ε *can be experimentally determined by measuring the maximum difference in osmolite aqueous concentrations -between the internal core (*C_T_^C^*) and the external environment (*C_T_^E^*)- that vesicles can bear. For oleic acid, *ε *was found equal to 0.21 [27]. In our model, at the critical bursting point, vesicles are assumed simply to break down, releasing all their internal content into the external environment and remaining in solution as flat bilayers, since bilayer sealing processes are not considered in this model. On the other hand, deflated vesicles are supposed to be able to divide in order to minimize the bending energy. The dividing condition is reached when they can form two equal volume spherical daughters (*ϕ *= 2^1/3^). So *η *introduces a tolerance that is linked to the relative flexibility exhibited by any membrane. However, as a simplifying assumption in this work, *η *= 0 will be supposed. As already mentioned, after each division, all the molecular content of the mother vesicle is halved in the deterministic approach, while it is randomly distributed between the twin daughters in the stochastic simulations.

### The Ribocell model

Figure 1 reports the internal metabolism proposed for the Ribocell. Both pairs of RNA strands reversibly associate (1) and these processes are shifted towards the dimer formation and are strongly dependent on temperature. The replication of any RNA strand is catalyzed by the polymerase R_P _according to the steps in bracket (2). This process is described as a catalytic template-directed addition of mononucleotides with high fidelity and processivity. It starts with R_P _binding any of the monomeric strands S (S = R_P_, _c_R_P_, R_L _and _c_R_L_) to form the complex R@S. This complex will then initiate the polymerization of the conjugate strand _c_S, by coupling and iteratively binding the complementary bases and releasing the by-product W. When the strand _c_S has been completely formed, the polymerase ribozyme releases the new dimer. Finally, the ribozyme R_L _catalyzes the conversion of the precursor P into the lipid L (3).

Table 1 shows the values of all kinetic constants and membrane permeabilities used in this work, with their respective references. In this work, the length of the two ribozymes is assumed to be 100 nucleotides long (20 being the minimum base number for observing a folded RNA conformation). Both R_L _and R_P _are created with a random sequence of bases, and they are assumed to have similar kinetic behaviors for the sake of simplicity. The kinetic constants of formation *k*_SS _and dissociation *k*_S _of both dimers were set equal to the values experimentally measured for a sequence of 10 nucleotides [28]. The kinetic constants for both complex formation R@S and complex dissociation R@S_c_S (S = R_P_, _c_R_P_, R_L _and _c_R_L_) were set equal to those measured for the human enzyme β-polymerase [29] that binds a DNA single strand and dissociates from a DNA double helix, respectively. The rate constant for the catalytic synthesis of lipid *k_L _*was taken to be 10^5 ^times larger than that of the splicing reaction, catalyzed by the hammerhead ribozyme [30]. The kinetic behavior of different nucleotides is assumed to be the same and a single value is assigned to *k*_NTP _derived from experimental data simulations (De Frenza private communication) of the DNA template directed synthesis in fatty acid mixed vesicles [31]. In the same way, the common value of the membrane permeability to activated nucleotides was also estimated, while the membrane is assumed impermeable to genetic material. The kinetic constants of the membrane/aqueous solution lipid exchange for oleic acid vesicles are taken from a previous work [26] where they were obtained by simulating the competition between isotonic and osmotically swollen oleic vesicles [27]. The amphiphile head area *α_L_* = 0.3 nm^2 ^and the osmotic tolerance ε = 0.21 are defined according to data reported in literature for oleic acid vesicles [27]. The only two parameters assigned arbitrarily are therefore the membrane permeability to the byproduct: *P*_W _= 0.0 cm/s, based on the assumption that W is a charged species, and the permeability to the precursor: *P*_P _= 0.42·10^-8 ^cm/s, corresponding to the oleic acid membrane's permeability to Arabitol [32] and comparable to those of similar organic compounds.

A common simplifying assumption to both approaches is to consider the external concentrations of nucleotides (NTPs), lipid precursor (P) and inert compound (B) to be constant throughout the time-courseof the process, thanks to an incoming flux of material in the reactor vessel: *continuous stirred tank reactor approximation*. The byproduct (W) concentrations is also assumed to be constantly equal to zero outside. For all these compounds, except B, the internal aqueous concentration is zero at the beginning. Moreover, all the calculations are performed starting from an initial isotonic condition, so that the internal concentration of B is properly adjusted in order to counterbalance the presence of the lipid precursor and nucleotides outside and the genetic material inside, respectively.

As has been pointed out before, given the set of kinetic parameters reported in Table 1 we are looking for a set of initial concentrations that can allow the Ribocell to reach a stationary regime of growth and division, i.e. a dynamic state where the protocell grows and divides producing two twin daughters the same size as their mother at the beginning of its life cycle.

In order to achieve this in our model, a spontaneous synchronization between membrane and the aqueous volume core of the self-replicating vesicle must take place. By introducing *the control growth coefficient*γ as the ratio between the relative change rates of volume *v*_V _and surface *v*_S_:

(4) $$\gamma =\frac{v_{V}}{v_{S}}=\left(\frac{1}{V_{C}}\frac{dV_{C}}{dt}\right)/\left(\frac{1}{S\mu }\frac{dS\mu }{dt}\right)$$

it is easy to show(unpublished observations) for growing protocells that synchronization can take place only if *γ *= 1, while if *γ *> 1 the volume will increase much faster than the membrane surface and the vesicle can become energetically unstable, leading to an osmotic burst. On the other hand, if *γ *< 1 then the vesicle will divide decreasing in size generation by generation. It is beyond the scope of this work to go into the mathematical details of this formula, so the interested reader should refer to an incoming paperfor a more detailed discussion.

Nevertheless, it is worthwhile to keep in mind that *v*_S _is essentially proportional to the rate of lipid synthesis, since amphiphile uptake by the membrane is very fast when the concentration of lipids is above the equilibrium value. Instead, having assumed the external value *C*_T_^E ^to be constant and a vesicle being in a osmotic balanced condition: *C*_T_^E^≈ *C*_T_^C ^= *V*_C_*N*_T_^C^,*v*_V _is driven by the overall internal *N*_T_^C ^population rise. Therefore, since *N*_T_^C ^increases essentially owing to the waste production that takes place with ribozyme self-replication and lipid synthesis, this has the effect of coupling the membrane reproduction with the genome replication and with the volume growth: *osmotic synchronization*.

**Figure 2:**
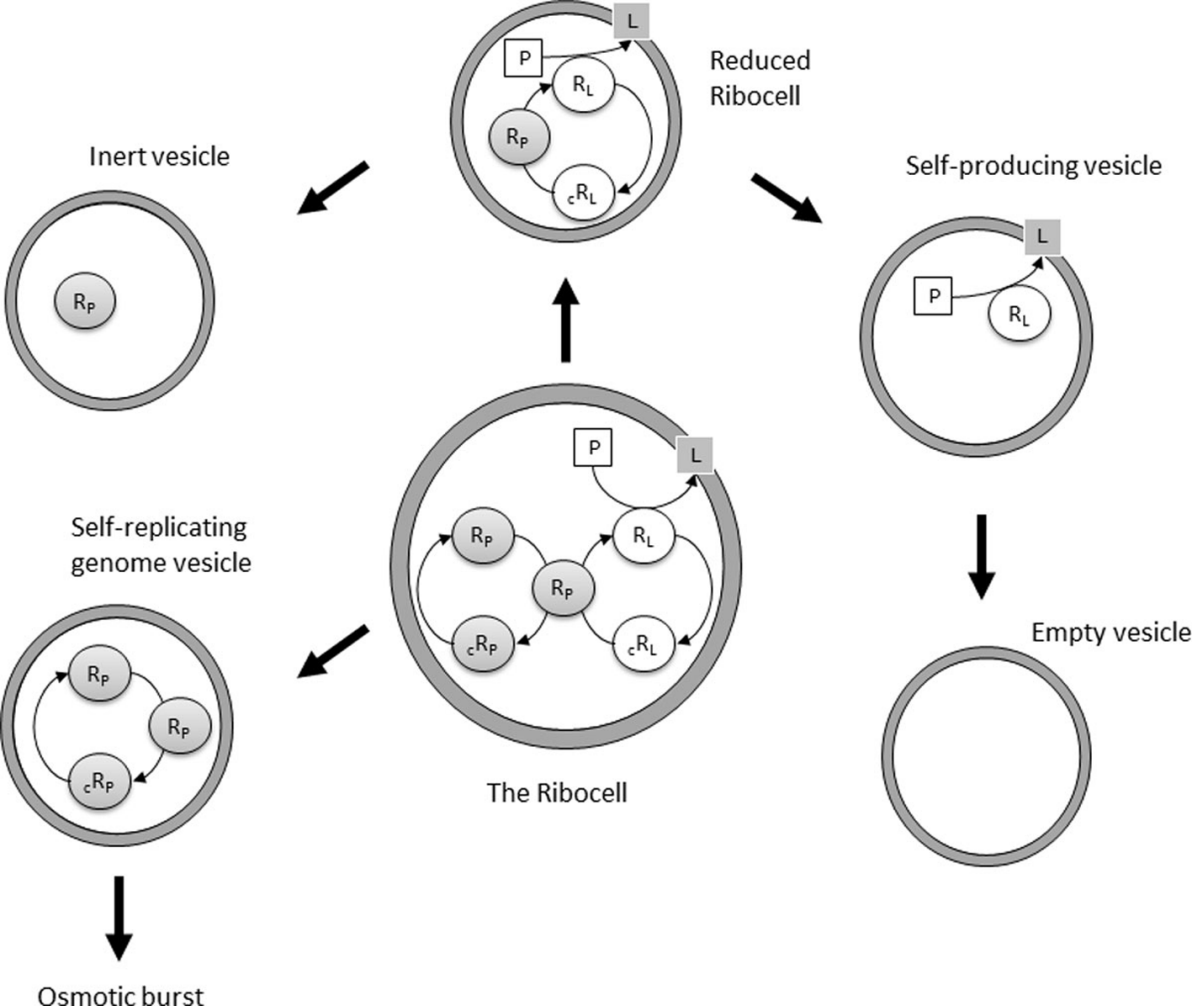
**Different-reacting protocells and vesicles obtained by RNA segregation due to Ribocell division**. Nucleotides (NTPs) and waste (W) have been omitted for the sake of clarity, along with the reversible association of RNA.

When the Ribocell reaches a stationary regime, at each division the genetic materials can be randomly distributed between the daughters. If the amount of genetic material is very low, then this can result in a separation of R_P _from the other RNA strands. In fact, the Ribocell must contain a minimum genetic kit of three RNA filaments in order to be capable of self-replicating its entire genome: one R_P _that catalyzes the RNA base pair transcription, one (R_L _or _c_R_L_) and one (R_P _or _c_R_P_) that work as templates for the transcription. Moreover, since R_L _is necessary to catalyze lipid precursor conversion, the optimal minimum 3-ribozyme kit must be made up of 2R_P _and one R_L_. This minimum kit should be at least doubled before cell division, in order to have a chance that both daughters continue to be active. Therefore, if a random distribution of RNA filaments takes place after vesicle division, ribozyme segregation between the two daughters might occur. Different scenarios can be envisaged as sketched in Figure 2: *death by segregation *is reached if vesicles are produced without any ribozymes (*empty vesicles*) or containing one lone R_P _or many filaments of _c_R_P _and/or _c_R_L _(*inert vesicles*). Vesicles that encapsulate R_L _strands are *self-producing*: they are able to synthesize lipids and then can grow and divide producing in turn self-producing and/or empty vesicles. On the other hand, vesicles containing more than one molecule of R_P _or both R_P _and _c_R_P _filaments are able to self-replicate this reduced genome (*self-replicating genome vesicles*) but they cannot self-reproduce the membrane. So they are destined for an osmotic burst due to an unbalanced increase in waste concentration. Finally, a reduced version of the Ribocell consists in a lipid aggregate that contains one R_P _filament and R_L_/_c_R_L _strands. As a consequence of this, *reduced ribocells *are able to replicate the R_L_/_c_R_L _genetic stuff, and at the same time to synthesize lipids. Therefore, they can grow and divide, producing in turn at least one reduced ribocell and/or self-replicating, inert and empty vesicle. With the help of stochastic simulations, we will try to explore all the possible scenarios.

## Results and discussion

### Deterministic analysis

In the present paper, we firstly explore the dependence of the stationary regime on the external concentrations of substrates for ribozymes 100 nucleotides long setting *k*_L _to 1.7·10^3^s^-1^M^-1^, i.e. the minimum value in the previously observed synchronization range. The aim of this preliminary deterministic study is to find the optimal initial conditions in order to achieve a stationary regime with the shortest life time. All the outcomes are reported in additional file 1.

**Figure 3:**
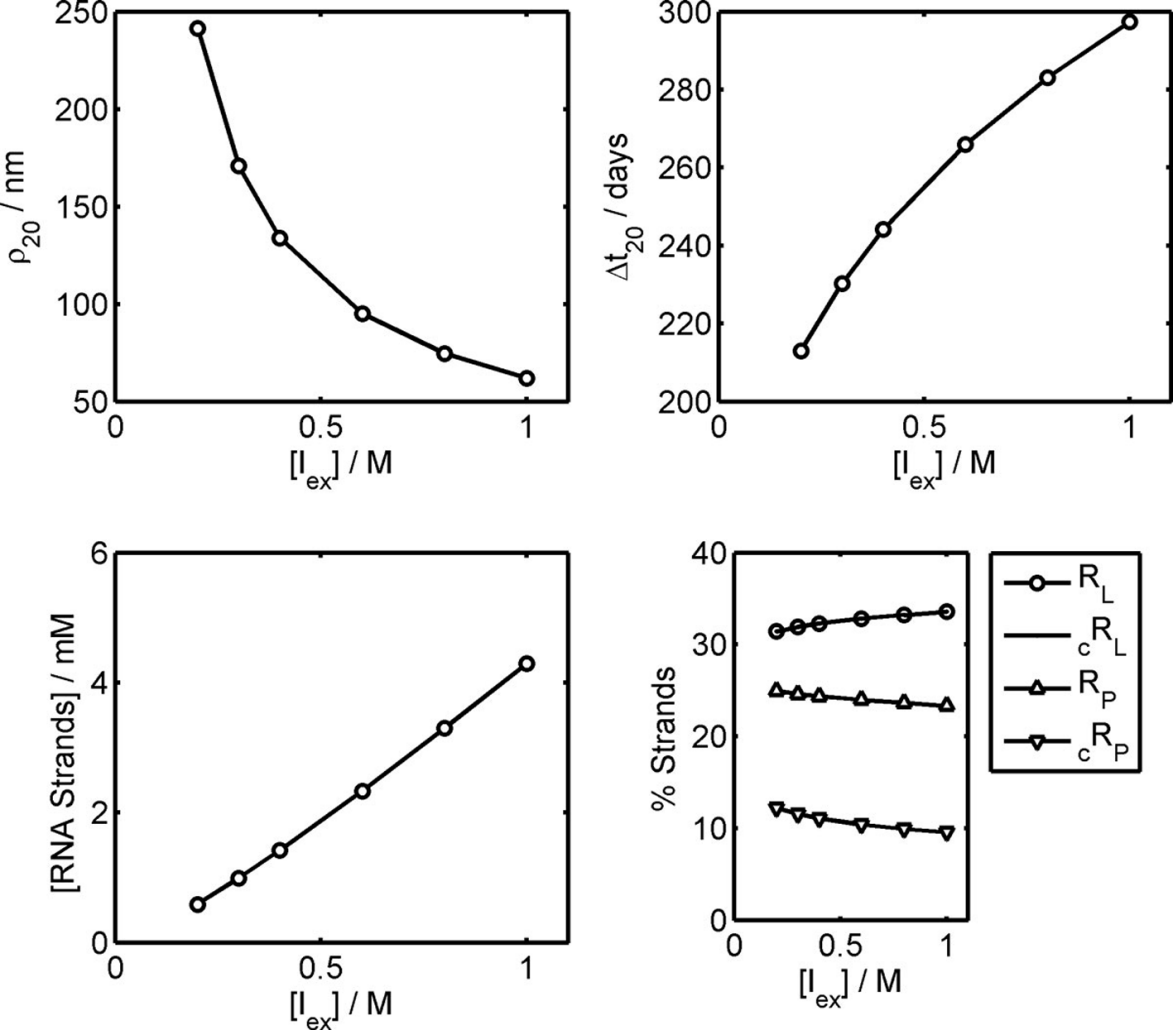
**Dependence of the Ribocell stationary regime on the external concentration of the inert compound [I_ex_]**. Vesicle radius (left upper plot), division time (right upper plot), overall internal concentrations of RNA strands (left lower plot) and genome composition percentage (right lower plot) were determined after 20 generations.

As first, the dependence of Ribocell state on overall external concentration *C*_T_^E ^is analyzed at the stationary regime, reached after 20 generations. Since [*P*_ex_] = [*N*_ex_] = 5.0·10^-4^M, the overall external concentration can be approximated to *C*_T_^E^≈[*I*_ex_]. The upper plots in Figure 3 show that when the overall external concentration [*I*_ex_] increases, then the Ribocell radius *ρ*_20 _decreases, while the life cycle Δ*t*_20 _rises. Thus, vesicles become smaller and more dormant as the overall external concentration rises. This can be ascribed to the mechanism of synchronization itself and is in agreement with what we reported in a recent work (unpublished paper) where an inverse dependence of the vesicle steady size on overall external concentration was explicitly derived from the general stationary condition *γ *= 1. On the other hand, the observed increase in Ribocell life time is a direct consequence of the reduction in size, since a smaller membrane surface decreases the transport efficiency of substrates (lipid precursor and nucleotides) from outside. As a consequence of this, all metabolic processes slow down since they are sustained by the transport of external substrates. These two effects, i.e. the increase in lifetime and the slowdown of the metabolism, determine the linear rise in concentration of the overall genetic material, see the lower left plot of Figure 3. At the steady regime, the genome composition is almost independent of [*I_ex_*], as shown by the lower right plot of

Figure 3. Two equal fractions of R_L _and _c_R_L _strands are present, both around 33%, while the percentages for R_P_, and _c_R_P _are lower: 24% and 10%, respectively. Due to the high *k*_ss _value, the ribozymes are mainly present inthe form of dimers and this accounts for the equal fractions observed for the lipase ribozymes, while the percentage of R_P _is greater than that of _c_R_P_, since some R_P _strands are involved as catalyzers in template duplication. This also explains why the overall percentage of polymerase ribozymes (~34%) is lower than that of lipase ribozymes (~66%) in fact, not all polymerase ribozymes are available as templates for duplication. Moreover, this asymmetry is amplified as long as the total concentration of the genetic material increases, see data in additional file 1.

**Figure 4:**
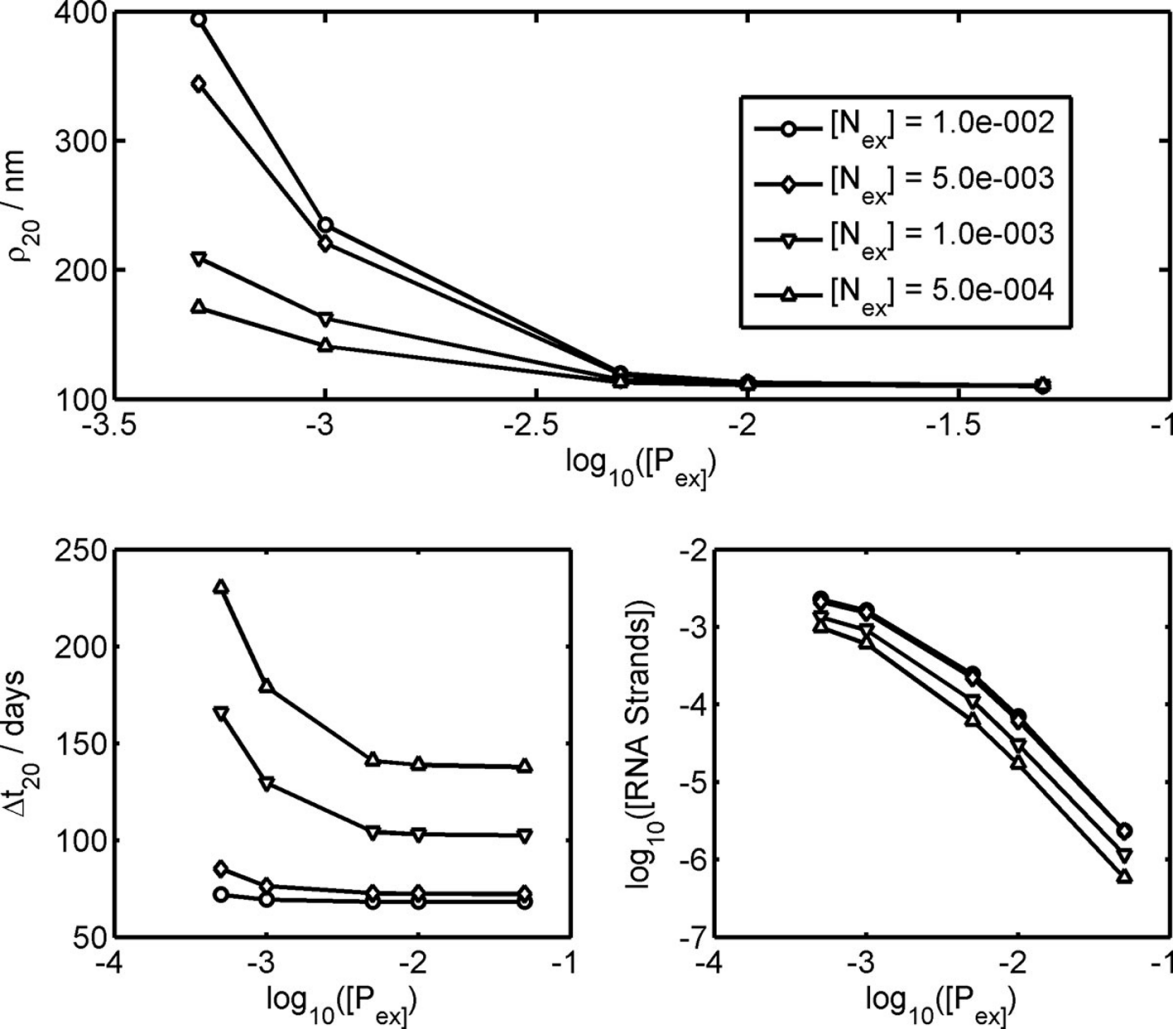
**Dependence of the Ribocell stationary regime on the external concentration of nucleotides [N_ex_] and lipid precursor [P_ex_]**. Vesicle radius (upper plot), division time (left lower plot) and logarithm of the overall internal concentrations of RNA strands (right lower plot) were determined after 20 generations.

Setting [*I*_ex_] = 0.3 M, we study the dependence of the stationary division regime on the external concentration of the substrates: lipid precursor [*P*_ex_] and nucleotides. The same concentration value [*N*_ex_] is set for the four different nucleotides since they have been assumed to have the same kinetic behavior. The upper plot of Figure 4 shows the opposite effects of [*N*_ex_] and [*P*_ex_] on the stationary vesicle radius *ρ*_20_. Higher [*N*_ex_] concentrations speed up genome self-replication with respect to lipid synthesis, accelerating waste production and leading to larger core volumes. Conversely, increasing [*P*_ex_] reduces *ρ*_20 _since membrane self-reproduction becomes faster. For the same reason, the total concentration of genetic material is increased due to the high concentrations of nucleotides and the low concentrations of the lipid precursor, while, both substrate concentrations decrease cell life time when they are increased, since all the metabolic processes are accelerated. If [*N*_ex_] ≥ 0.05 M, the Ribocell undergoes an osmotic burst since volume growth is too fast compared to lipid production for any value of [*P*_ex_] in the studied range (see additional file 1).

**Figure 5:**
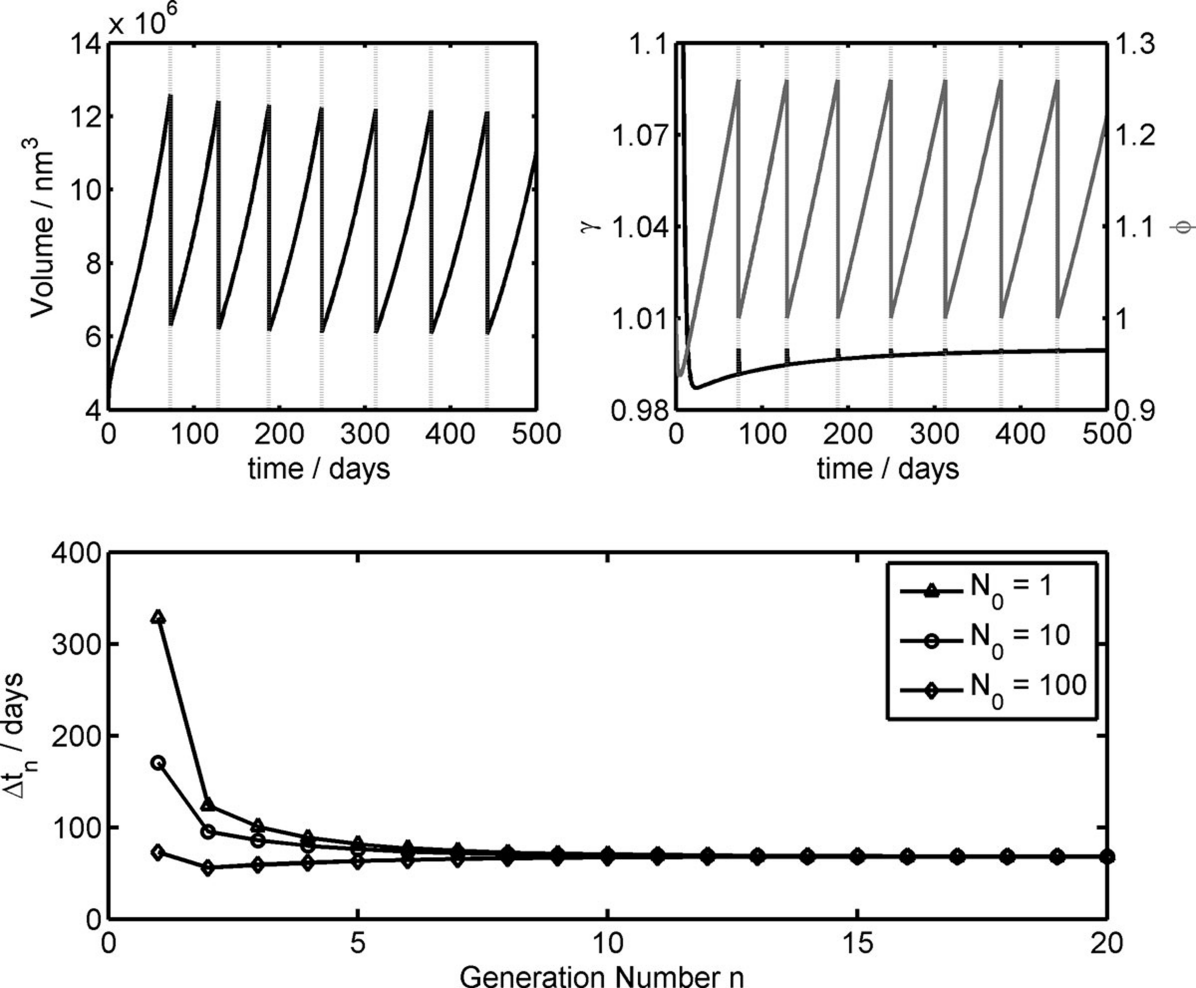
**Deterministic time evolution of the Ribocell**. Aqueous core volume (left upper plot), growth control coefficient *γ*, on the left axis, and reduced surface *ϕ*, on the right axis,(right upper plot) are reported against time for a ribocell starting with the initial number N_0 _of both R_c_R_L _and R_c_R_P _dimers equal to 100; on lower plot, division times against generation number for different initial N_0 _values are displayed. In the upper plots, the vertical dashed lines represent the cell division times that take place when *ϕ *= 2^1/3^.

In the upper plots of Figure 5, the time trends of the core volume on the left, the growth control coefficient *γ *and the reduced surface *ϕ *(on the right) are reported for a Ribocell starting with a number of dimers N_0 _= 100 for both R_c_R_L _and R_c_R_P_. These plots show that after a few generations the steady division regime is reached, as confirmed by *γ* that tends to 1.0. At the real beginning, the Ribocell undergoes a fast volume increase, as demonstrated by *γ *> 1.0 and *ϕ *< 1.0. This is due to the transport process of the lipid precursor and the nucleotides from the external environment that blows up the vesicle. In the lower plot of Figure 5, the division time is reported against the number of generations for ribocells starting with a different initial number of strands N_0_. In all three cases, the same division time is reached after 10 generation:s, i.e. 68.2 days, that remains constant for the following generations. The higher N_0_, the faster the cell division in the first generations.

**Figure 6:**
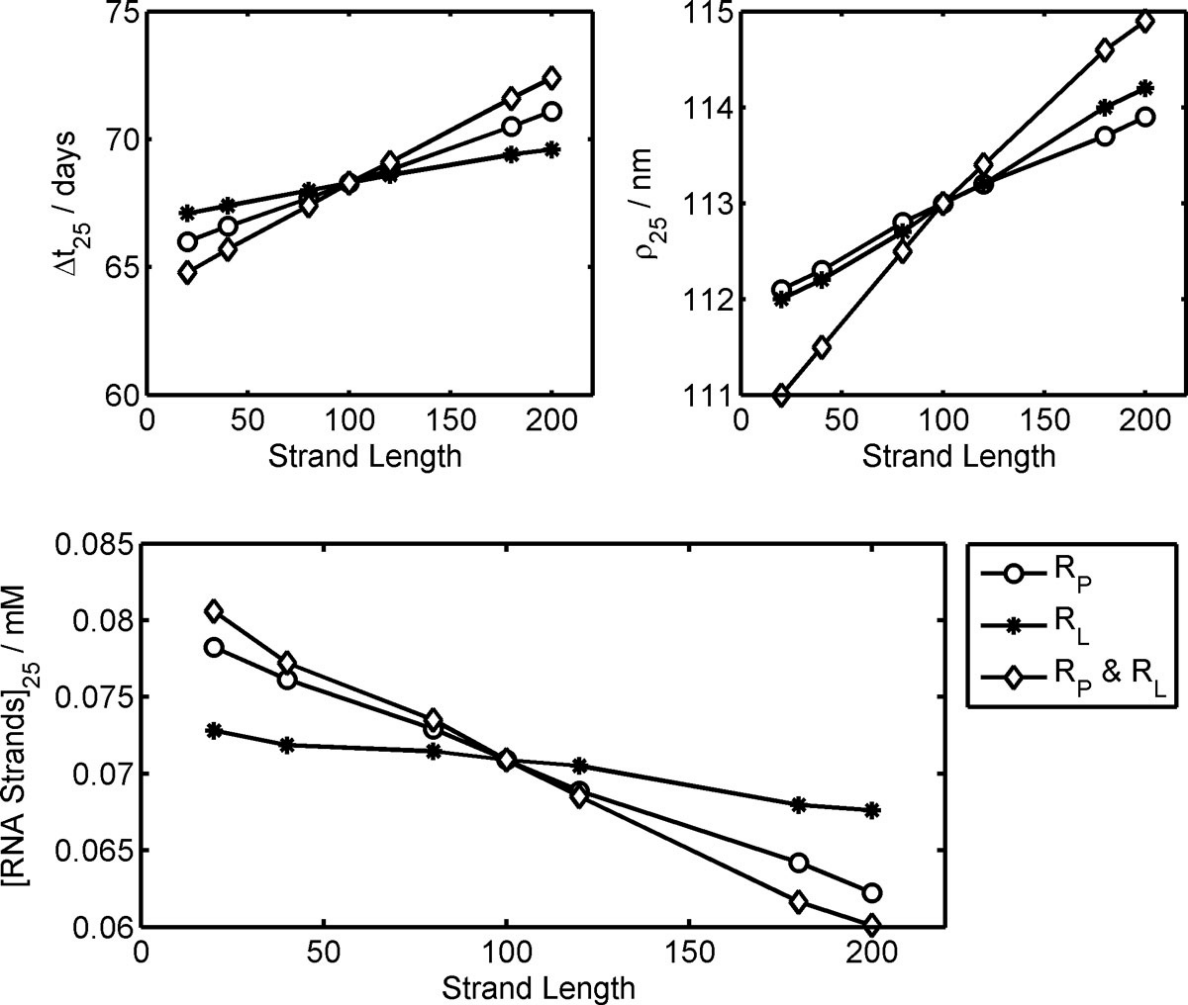
**Influences of ribozyme length on the Ribocell stationary regime**. Life time Δ*t*_25_, radius ρ_25 _and RNA total strand concentration [RNA Strands]_25 _after 25 generations are reported against ribozyme length. The legend reports the ribozymes that are changed in size.

Having determined optimal external conditions, the influence of ribozyme length is now investigated by keeping all the other kinetic parameters constant. Calculations have been performed changing in turn the length of R_L _or R_P_, and fixing the size of the other ribozyme to 100 bases, or changing the size of both R_L _and R_P _but keeping the same length. Results are reported in Figure 6. In all the studied cases, an increase in strand length determines at the stationary regime Ribocells with a longer life cycle Δ*t*_25_, with a larger radius ρ_25 _and a lower RNA total concentration [RNA Strands]. The variations observed are quite small compared to those for 100-base ribozymes, except for the [RNA Strands] that show a change about 15%. Furthermore, the Ribocell shows to be much more sensitive to the change in size of the polymerase ribozyme R_P _rather than R_L_. This can be ascribed to the fact that, being longer, R_P_, requires more time to self-replicate and this decreases the overall concentration of all the polymerase ribozymes and in turn the efficiency of genome self-replication and membrane self-reproduction.

**Figure 7:**
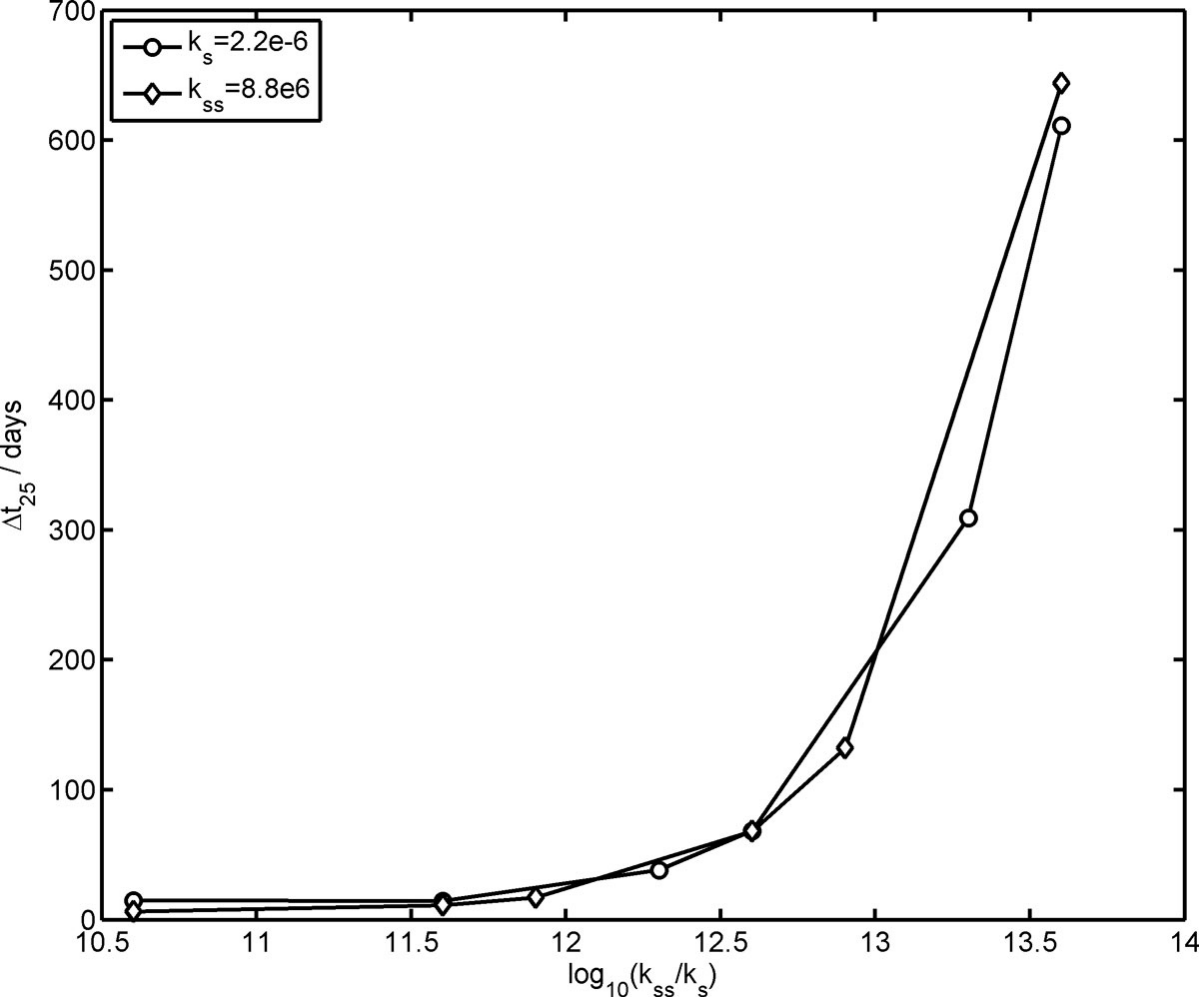
**Influences of *k*_ss _and *k*_s _kinetic constants on the Ribocell stationary regime**. Life time Δ*t*_25 _after 25 generations is reported against the log_10 _of the thermodynamic constant of RNA dimerization. The legend displays the value of the kinetic parameters kept constant.

Finally, Figure 7 displays the dependence of Δ*t*_25 _on the kinetic constants for RNA dimer formation *k*_SS _and dissociation *k*_S_. The plot clearly shows that the Ribocell life cycle at stationary regimes does not depend explicitly on the kinetic constant single values *k*_SS _and *k*_S _but on their ratio: *k*_SS_/*k*_S_, that is on the thermodynamic constant of RNA dimerization. The more thermodynamically stable the RNA dimers, the longer it takes to observe Ribocell self-reproduction. For instance, if *k*_SS_/*k*_S _is decreased by two orders of magnitude, the Ribocell life time reduces from 68.2 days to 11.8-6.4 days. The study of Ribocell time behavior approaching the stationary regime as a function of *k*_SS _and *k*_S _values would require a much deeper analysis that is beyond the scope of this paper.

### Stochastic simulations

Stochastic simulations were performed by means of the parallel version of ENVIRONMENT, running 32 statistically equivalent simulations of a 10-ribocell solution on different CPUs. Therefore, the outcomes were obtained as averages from a population of 320 vesicles. Kinetic parameters used for simulations are those reported in Table 1 while the initial conditions are shown in bold by additional file 1. At each cell division, only one of the two offspring was kept while the other was discarded in order to reduce computation time, thus keeping the number of monitored vesicles constant. This is in agreement with the assumption that the external concentrations of all substrates are fixed due to an incoming flux of material, i.e. the substrates cannot ever be exhausted.

**Figure 8:**
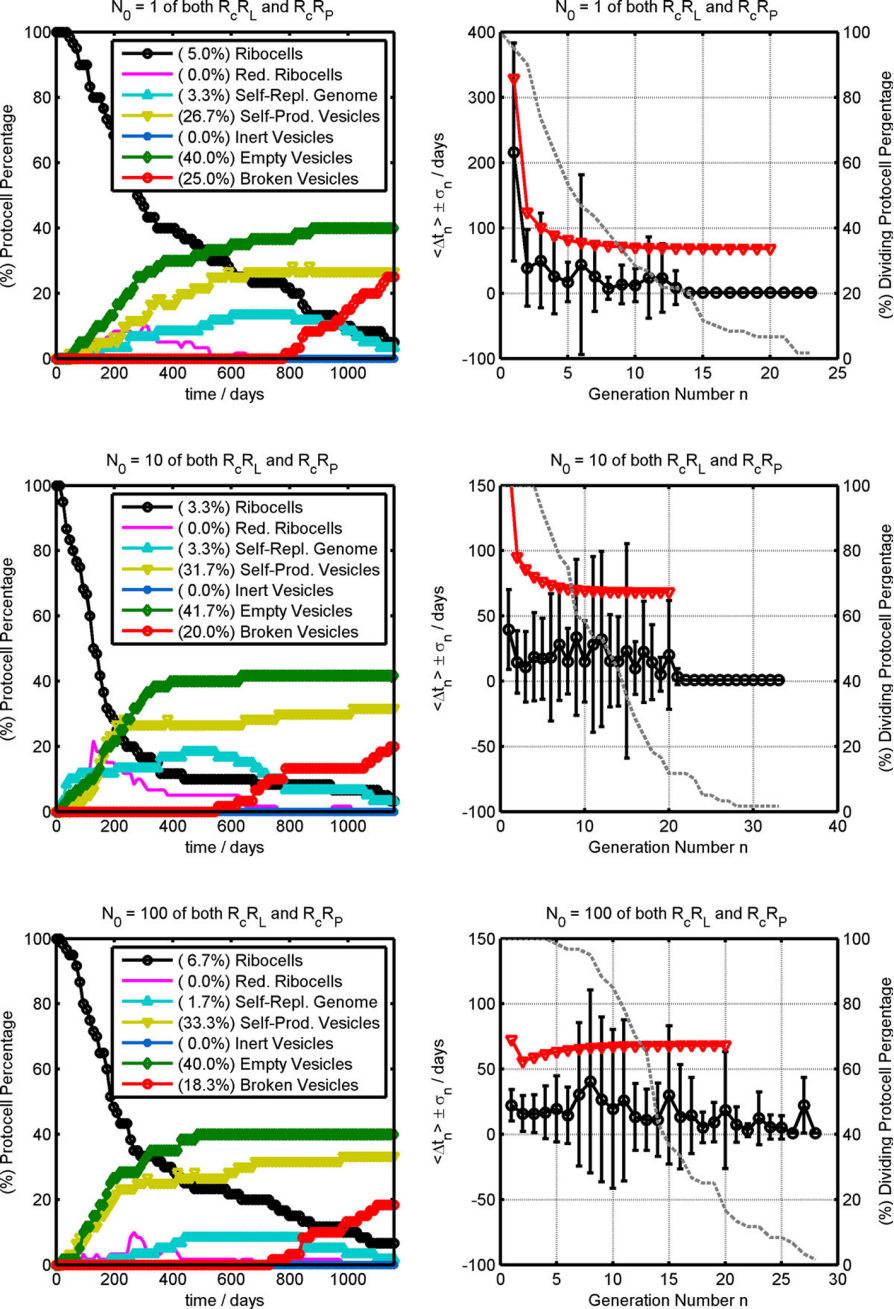
**Stochastic simulation outcomes**. Plots on the left show the time evolution of ribocell populations while the legend shows the final compositions; plots on the right report the stochastic <Δt_n _> (circles with error bars) and deterministic Δt_n _(red triangles) division times on the left axis while the percentage of dividing protocells (dashed gray lines) is reported on the right axis against the generation number *n*. The initial genome was by made up as follows: 1 (upper plots), 10 (middle plots) and 100 (lower plots) dimers of both R_c_R_L _and R_c_R_P_.

On the left in Figure 8, the time evolutions of protocell populations obtained by stochastic simulations are reported for the three studied cases starting with a genome made up of 1, 10 and 100 dimers of both R_c_R_L _and R_c_R_P_. At the end of the simulations of all three cases, similar compositions of the protocell population are obtained with low percentages of real ribocells (3.3-6.7%) while the most populated fractions are those of empty (40.0-41.7%) self-producing (26.7-33.3%) and broken (18.3-25.0%) vesicles, respectively. Reduced ribocells are present only in the first generations since they very soon decay into self-producing and empty vesicles. Inert vesicles, i.e. vesicles entrapping free chains of _c_R_P _and/or _c_R_L _or a single R_P_, are not formed and this can be ascribed to the high stability of RNA dimers and complexes so that the chance of finding free RNA monomers at the time of vesicle division is extremely improbable. Thus, the three studied cases are differentiated by their time behavior rather than by the final protocell population, as confirmed by the plots on the right in Figure 8, where the average division time <Δt_n _> and the number of dividing protocells are reported against the generation number. In agreement with deterministic predictions, for the first generations the average division times <Δ*t*_n _> are higher for ribocells starting with a lower initial number *N*_0 _of dimers although, in all cases, the deterministic Δ*t*_n _(red triangles) are greater than the stochastic averages (black circles with error bars). This can be partially ascribed to the fact that the average <Δ*t*_n _> is calculated on all the protocells that undergo the *n*-th division and only some of them are real ribocells. In fact, generation by generation, the protocell population is enriched by self-producing vesicles that can divide more quickly if a free R_L _monomer is present and this lowers the average division time.

**Figure 9:**
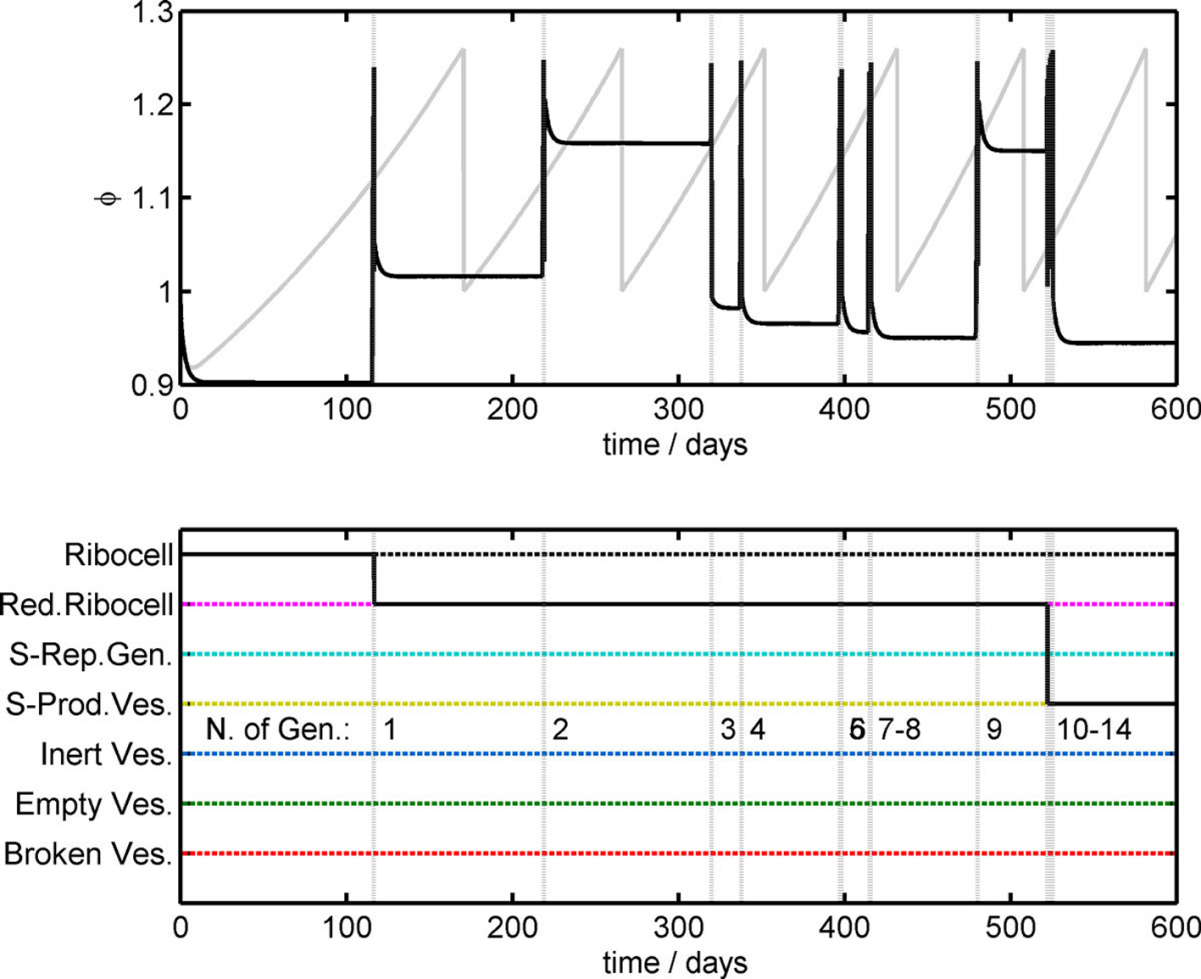
**Stochastic time behavior of a single ribocell with *N*_0 _= 1**. In the upper plot, the stochastic time trend (black line) of the reduced surface *ϕ *is compared with the deterministic time course (gray line); vertical dashed lines indicate the simulated division times. The lower plot explicitly shows the 14 divisions and the two transformations of the ribocell due to RNA segregation. After the first division, it becomes a reduced ribocell and after 10 generations a self-producing vesicle.

As an example, Figure 9 reports the time behavior of a single Ribocell with a starting genome made up of just one R_c_R_L _and one R_c_R_P_. In the upper plot, a comparison between the reduced surface time course determined deterministically and simulated stochastically is shown. As can be seen, the stochastic time trend presents a very irregular time behavior compared to the deterministic one that describes a highly synchronized oscillating regime of growth and division. In contrast, stochastic simulations highlight the alternation of dormant phases, where the reduced surface remains practically constant, both the core volume and the membrane surface being constant (data not shown), to very active steps where protocell growth takes place very fast, leading to a division event.

**Figure 10:**
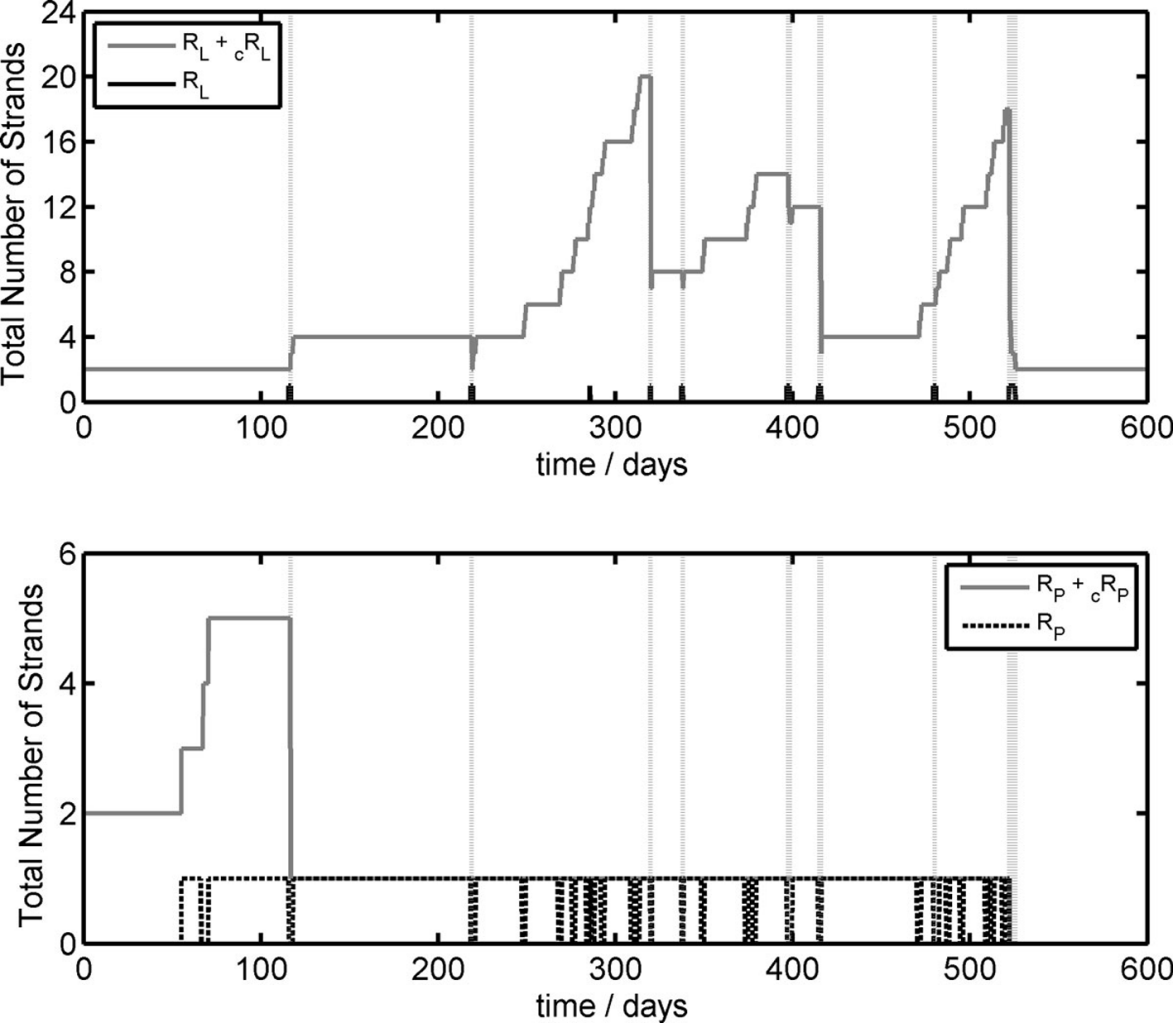
**Stochastic time evolution of the genome composition of a single ribocell with *N*_0 _= 1**. In the upper plot, the total number of lipase strands (gray lines) and the R_L _free monomers (black dashed lines) are reported against time, while in the lower plot the total number of polymerase strands (gray lines) and the R_P _free monomers (black dashed lines) time courses are shown.

In order to account for this behavior, in Figure 10 the time course of the total number of lipase R_L _and polymerase R_P _ribozymes present as monomers, complementary strands, dimers and complexes, are reported against time, along with the number of free R_L _and R_P _monomers that exhibit catalytic activity. As can be seen, the fast growth and division step corresponds to the presence in the vesicle core of a free R_L _chain while, in the dormant phase, ribozymes are all coupled in the form of dimers or complexes. In fact, during RNA template transcription in the first generation life time, the volume remains practically constant since the amount of waste molecules produced is not sufficient to promote a substantial water flux from the external environment. As a consequence, self-producing vesicles with a genome made up only of R_L _monomers can reproduce very efficiently since no dormant phase can occur, given that the formation of R_c_R_L _dimers is impossible. This is what happens at high generation numbers in the protocell population time evolutions reported in Figure 8. It is clear in both cases with a starting genome *N*_0 _equal to 1 and 10. In fact, at high generation numbers, the only dividing protocells are self-producing vesicles that present free R_L _monomers in the core volume. Although there are very few of these protocells, they can divide very efficiently, with a Δ*t *of around 0.81 days, and with division times that are very close to one another, so the population average <Δ*t*_n _*>*seems very low with a small error bar as shown by the upper and middle plots on the left in Figure 8.

## Conclusions

In this paper, we applied an already published Ribocell *in silico *model [15,16] to the case of two hypothetical ribozymes 100 nucleotides long by using the kinetic parameters reported in Table 2. The kinetic constant for the lipid formation, *k*_L_, was set equal to 1.7E+3s^-1^M^-1^, the lowest value that exhibited a stationary regime of growth and division in a previous work [16] in which ribozymes 20 nucleotide long were assumed. Length of 100 nucleotides was chosen as a compromise between the need to reduce calculation times and the choice of a plausible ribozyme size, keeping in mind that the RNA subunits in ribosomes devoted to protein elongation have a comparable length [18,19].

By means of deterministic analysis, the robustness of the stationary regime was also investigated as a function of the initial conditions, the length of ribozymes and the kinetic constants of the RNA dimerization. For 100-base long ribozymes, the best experimental conditions in terms of the external concentrations have been found: [*N*_ex_] = [*P*_ex_] = 1.0E-2M and [*I*_ex_] = 0.3M in order to observe a stationary regime with the lowest division time: Δ*t*_20_ = 68.3 days. A so high protocell life time can be mainly ascribed to the RNA reversible association that is shifted towards the dimer formation rendering the concentration of the catalyzers R_L _and R_P _very low. This has been confirmed by analyzing the dependence of the life cycle on the thermodynamic constant of ribozyme dimerization. A small influence on the stationary regime was observed on changing the length of the RNA strands: an increase in filament size determines higher life times and a lower amount of genetic material. This effect is more pronounced when the R_P _length changes. On the other hand, the external concentrations of substrates and inert compounds appear to highly affect the stationary regime in terms of vesicle size, genetic material amount and genome composition. Moreover, deterministic calculations have also shown that the stationary regime can be reached from very different initial genome composition, although when too much free R_L _ribozymes are present at the beginning the death for ribozymes segregation can be observed since the protocell divides too quickly before the genome replication. Stochastic simulations have been done starting from a population of 320 identical ribocells with an initial genome composed by *N*_0 _= 1,10 and 100 dimers of both R_c_R_L _and R_c_R_P_. The analysis of simulations outcomes shows that the ribocell time behaviors is highly influenced by random fluctuations. Since the genetic material is randomly distributed at each cell division, this can produce different type of protocells, ranging from empty vesicles to genuine ribocells, their internal metabolism being highly influenced by the presence of the catalytic RNA strands. In fact, deterministic analysis cannot take into account the disappearance of ribozymes due to a vesicle division, since this approach simply halves the genetic amount and follows the reacting molecule time courses in terms of population averages, i.e. real positive numbers that can be less than one without being zero. On the other hand, the stochastic simulations are more realistic to the random loss of ribozymes from the genome being capable of describing a population of protocells with completely different time behaviors. As a consequence, the simulation outcomes show that ribocells are not enough robust to survive to random fluctuations. In fact only about the 5% of the initial population survive as genuine ribocells after 15-25 generations and on a longer time window they are destined for extinction. Furthermore, the time course of each single protocell is also greatly influenced by intrinsic stochasticity in particular by the time fluctuations of the RNA dimer dissociation. In fact, when all the RNA strands are associated in dimers, protocells remain in a lazy phase, whereas free R_L _monomers induce fast growth and division steps and free R_P _cause the fast RNA replication without changing the vesicle size appreciably. Therefore these two process are synchronized only by chance and this also represents a reason of weakness of this model protocell.

In order to implement experimentally ribozymes-based minimal cells two main improvements are necessary. As first, more free monomers of both R_L _and R_P _must be available in the vesicle core so that the ribocell life cycle will be speeded up and the division time lowered. This can be achieved by increasing the working temperature since it has been recently show that fatty acid vesicles are stable up to 90°C [33] and the efficiency of the self-catalyzed replication of RNA strands increases with a temperature rise. This is also in agreement with our results since the *k*_SS _value used can be considered as the appropriate 100-base long RNA association constant for a higher temperature than 25°C [34]. For a more detailed theoretical analysis at high temperature, it is necessary, of course, to estimate the kinetic constants for all the steps involved in the internal metabolism. This will be the topic of a future work. The second necessary improvement consists in finding a way to really synchronize the genome self-replication and the membrane reproduction. This is a much more complex task to achieve. Working with high concentration of the genetic material, it can avoid, or at least reduce, the ribozymes segregation and this should be compatible with a high working temperature. The best strategy could be to have a fine control of the R_c_R_L _dissociation since when free R_L _monomers are present in the aqueous core the membrane growth quickly and the division takes place very soon. Thus the R_c_R_L _dissociation can act as a trigger for the membrane growth and division.

Finally rephrasing the George Box famous sentence, we are aware that this *in silico *Ribocell model is in a some way wrong, but we hope it might inspire researchers involved in the lab implementation of the ribozymes-based minimal cell.

## Competing interests

The authors declare that they have no competing interests.

## Supplementary Material

Additional file 1 Deterministic Outcomes of the Ribocell time behavior: stationary values for different initial conditions Click here for file
