# Supplementary material for: Stochastic simulations of minimal cells: the Ribocell model
Source: BMC Bioinformatics. 2012 Mar 28;13(Suppl 4):S10. doi: 10.1186/1471-2105-13-S4-S10 (PMC3303737; doi:10.1186/1471-2105-13-S4-S10)
Supplement: Additional file 1 — Deterministic Outcomes of the Ribocell time behavior: stationary values for different initial conditions [file 1471-2105-13-S4-S10-S1.docx]

| **Genome Initial composition** | | **[*I*_ex_]** | **[*P*_ex_]** | **[N_ex_]** | ***ρ*_20_** | **Δ*t*_20_** | **Total RNA strands** | **Genome stationary composition** | | | |
| --- | --- | --- | --- | --- | --- | --- | --- | --- | --- | --- | --- |
| **R_L_/_c_R_L_/R_c_R_L_** | **R_P_/_c_R_P_/R_c_R_P_** | **M** | **M** | **M** | **nm** | **days** | **numbers** | **% R_L_** | **% _c_R_L_** | **% R_P_** | **% _c_R_P_** |
| 0/0/1 | 0/0/1 | 0.2 | 5.0E-04 | 5.0E-04 | 241.5 | 213.0 | 20934 | 31.4 | 31.4 | 24.9 | 12.2 |
|  |  | 0.3 |  |  | 171.0 | 230.3 | 12500 | 31.9 | 31.9 | 24.6 | 11.6 |
|  |  | 0.4 |  |  | 134.0 | 244.1 | 8618 | 32.3 | 32.3 | 24.4 | 11.1 |
|  |  | 0.6 |  |  | 95.1 | 265.9 | 5070 | 32.8 | 32.8 | 24.0 | 10.4 |
|  |  | 0.8 |  |  | 74.7 | 283.1 | 3468 | 33.2 | 33.2 | 23.6 | 9.9 |
|  |  | 1.0 |  |  | 61.9 | 297.4 | 2574 | 33.6 | 33.6 | 23.3 | 9.6 |
| **0/0/1** *^a^* | **0/0/1** | **0.3** | 5.0E-2 ... 5.0E-4 | 5.0E-02 | Osmotic Burst | | | | | | |
|  |  |  | 5.0E-02 | **1.0E-02** | 110.5 | 68.3 | 8 | 25.0 | 25.0 | 25.0 | 25.0 |
|  |  |  | **1.0E-02** |  | **113.0** | **68.2** | **258** | **25.2** | **25.2** | **25.6** | **24.0** |
|  |  |  | 5.0E-03 |  | 120.2 | 68.4 | 1092 | 25.3 | 25.3 | 25.3 | 24.2 |
|  |  |  | 1.0E-03 |  | 235.0 | 69.4 | 54346 | 25.4 | 25.4 | 25.3 | 24.0 |
|  |  |  | 5.0E-04 |  | 394.3 | 72.0 | 358830 | 25.6 | 25.6 | 25.4 | 23.5 |
|  |  |  | 5.0E-02 | 5.0E-03 | 110.7 | 72.4 | 8 | 25.0 | 25.0 | 25.0 | 25.0 |
|  |  |  | 1.0E-02 |  | 112.9 | 72.5 | 226 | 25.7 | 25.7 | 25.2 | 23.5 |
|  |  |  | 5.0E-03 |  | 119.3 | 72.6 | 954 | 25.6 | 25.6 | 25.5 | 23.4 |
|  |  |  | 1.0E-03 |  | 220.9 | 76.3 | 41770 | 25.8 | 25.8 | 25.6 | 22.8 |
|  |  |  | 5.0E-04 |  | 344.3 | 85.4 | 216408 | 26.4 | 26.4 | 25.8 | 21.4 |
|  |  |  | 5.0E-02 | 1.0E-03 | 110.7 | 102.6 | 4 | 25.0 | 25.0 | 25.0 | 25.0 |
|  |  |  | 1.0E-02 |  | 111.9 | 103.2 | 108 | 26.9 | 26.9 | 26.9 | 19.4 |
|  |  |  | 5.0E-03 |  | 115.1 | 104.5 | 434 | 27.4 | 27.4 | 26.0 | 19.1 |
|  |  |  | 1.0E-03 |  | 162.6 | 129.7 | 10016 | 28.5 | 28.5 | 26.1 | 16.9 |
|  |  |  | 5.0E-04 |  | 209.5 | 166.0 | 31330 | 29.9 | 29.9 | 25.7 | 14.4 |
|  |  |  | 5.0E-02 | 5.0E-04 | Death by segregation | | | | | | |
|  |  |  | 1.0E-02 |  | 111.4 | 139.0 | 60 | 28.3 | 28.3 | 26.7 | 16.7 |
|  |  |  | 5.0E-03 |  | 113.2 | 141.1 | 228 | 28.9 | 28.9 | 25.9 | 16.2 |
|  |  |  | 1.0E-03 |  | 141.3 | 179.2 | 4364 | 30.4 | 30.4 | 25.5 | 13.7 |
|  |  |  | 5.0E-04 |  | 171.0 | 230.3 | 12500 | 31.9 | 31.9 | 24.6 | 11.6 |
| 0/0/1 | 1/0/1 | **0.3** | **1.0E-02** | **1.0E-02** | 113.0 | 68.3 | 258 | 25.2 | 25.2 | 25.6 | 24.0 |
| 0/0/1 | 5/0/1 |  |  |  | 113.0 | 68.2 | 258 | 25.2 | 25.2 | 25.6 | 24.0 |
| 0/0/1 | 10/0/1 |  |  |  | 113.0 | 68.1 | 258 | 25.2 | 25.2 | 25.6 | 24.0 |
| 0/0/1 | 25/0/1 |  |  |  | 113.0 | 68.0 | 260 | 25.0 | 25.0 | 25.8 | 24.2 |
| 0/0/1 | 50/0/1 |  |  |  | 113.0 | 67.8 | 262 | 25.2 | 25.2 | 25.6 | 24.0 |
| 1/0/0 | 2/0/0 |  |  |  | 113.0 | 68.3 | 258 | 25.2 | 25.2 | 25.6 | 24.0 |
| 5/0/0 | 2/0/0 |  |  |  | 113.0 | 68.7 | 254 | 25.2 | 25.2 | 25.6 | 24.0 |
| 10/0/0 | 2/0/0 |  |  |  | 112.9 | 69.0 | 254 | 25.2 | 25.2 | 25.6 | 24.0 |
| 25/0/0 | 2/0/0 |  |  |  | Death by segregation | | | | | | |
| 50/0/0 | 2/0/0 |  |  |  | Death by segregation | | | | | | |
| **0/0/10** *^a^* | **0/0/10** |  |  |  | **113.0** | **68.2** | **258** | **25.2** | **25.2** | **25.6** | **24.0** |
| **0/0/100** *^a^* | **0/0/100** |  |  |  | **113.0** | **68.2** | **258** | **25.2** | **25.2** | **25.6** | **24.0** |
